# The H7N9 influenza A virus infection results in lethal inflammation in the mammalian host via the NLRP3-caspase-1 inflammasome

**DOI:** 10.1038/s41598-017-07384-5

**Published:** 2017-08-08

**Authors:** Rongrong Ren, Shuxian Wu, Jialin Cai, Yuqin Yang, Xiaonan Ren, Yanling Feng, Lixiang Chen, Boyin Qin, Chunhua Xu, Hua Yang, Zhigang Song, Di Tian, Yunwen Hu, Xiaohui Zhou, Guangxun Meng

**Affiliations:** 1 Shanghai Public Health Clinical Center, Fudan University, Shanghai, 201508 China; 2 CAS Key Laboratory of Molecular Virology & Immunology, Institute Pasteur of Shanghai, Chinese Academy of Sciences, Shanghai, 200031 China; 3 Key Laboratory of medical virology, MOH/MOE, Shanghai Medical College, Fudan University, Shanghai, 200032 China; 4 Translational Medicine Research Center, Ruijin Hospital North, Shanghai Jiao Tong University School of Medicine, Shanghai, 201821 China; 5 Shanghai Chest Hospital, Shanghai Jiao Tong University, Shanghai, 200030 China

## Abstract

The avian origin influenza A virus (IAV) H7N9 has caused a considerable number of human infections associated with high rates of death since its emergence in 2013. As a vital component of the host innate immune system, the nucleotide-binding domain leucine-rich repeat containing receptor, pyrin domain containing 3 (NLRP3) inflammasome plays a critical role against H1N1 viral infection. However, the function of NLRP3 inflammasome in host immunological responses to the lethal H7N9 virus is still obscure. Here, we demonstrated that mice deficient for NLRP3 inflammasome components, including NLRP3, caspase-1, and Apoptosis-associated speck-like protein containing a CARD (ASC), were less susceptible to H7N9 viral challenge than wild type (WT) controls. Inflammasome deficiency in these animals led to significantly milder mortality and less pulmonary inflammation compared with WT mice. Furthermore, IL-1 receptor deficient mice also exhibited a higher survival rate than WT controls. Thus, our study reveals that the NLRP3 inflammasome is deleterious for the host during H7N9 infection in mice, which is due to an overwhelming inflammatory response via caspase-1 activation and associated IL-1 signal. Therefore, fine-tuning the activity of NLRP3 inflammasome or IL-1 signaling may be beneficial for the host to control H7N9 associated lethal pathogenesis.

## Introduction

In March 2013, the novel avian-origin influenza A virus (IAV) H7N9 was identified in China. H7N9 exhibits low pathogenicity in birds, but has an ability to bind certain mammalian cell receptors, and replicates at temperature close to the normal body temperature of mammals^1–3^. The infected patients often develop severe symptoms, including pneumonia and acute respiratory distress syndrome (ARDS)^4^. The H7N9 infection had caused 1230 confirmed human cases, with 428 deaths by 22 February 2017 [data from FAO (Food and Agriculture Organization of the United Nations)]. The severe symptoms and high mortality associated with H7N9 infection pose a big threat to the public health. Although recent studies of H7N9 have achieved great progress, clear definition of the host immunological responses to H7N9 is still lacking^1, 5, 6^.

Influenza virus infection mainly induces two types of innate immune responses^7^. A rapid antiviral response, i.e. type I interferon production, to facilitate intracellular control of viral replication; and a proinflammatory response characterized by secretion of cytokines and chemokines, which promotes recruitment of various immune cells for viral clearance^8^. As a key mediator of inflammation, the proinflammatory cytokine IL-1β plays a crucial role in the pathogenesis of various diseases^9^. The maturation and secretion of IL-1β require a protein complex called inflammasome, which activates caspase-1 to cleave proIL-1β to its mature form^10^. More importantly, caspase-1 and the NLRP3 inflammasome have been found essential for protecting mice against H1N1 IAV infection^11–13^.

In a previous study, we have demonstrated that H7N9 influenza virus isolated from human patients could establish successful infection of mice^14, 15^. Employing this murine model, in the current study we demonstrate a detrimental role for the NLRP3-caspase-1 inflammasome and associated IL-1 signal in H7N9 infection of mice. Our data suggest that dampening the NLRP3 inflammasome activity or inhibiting IL-1 signaling should be beneficial for the host during a lethal H7N9 challenge.

## Results

### H7N9 RNA induces NLRP3 inflammasome activation

**Figure 1:**
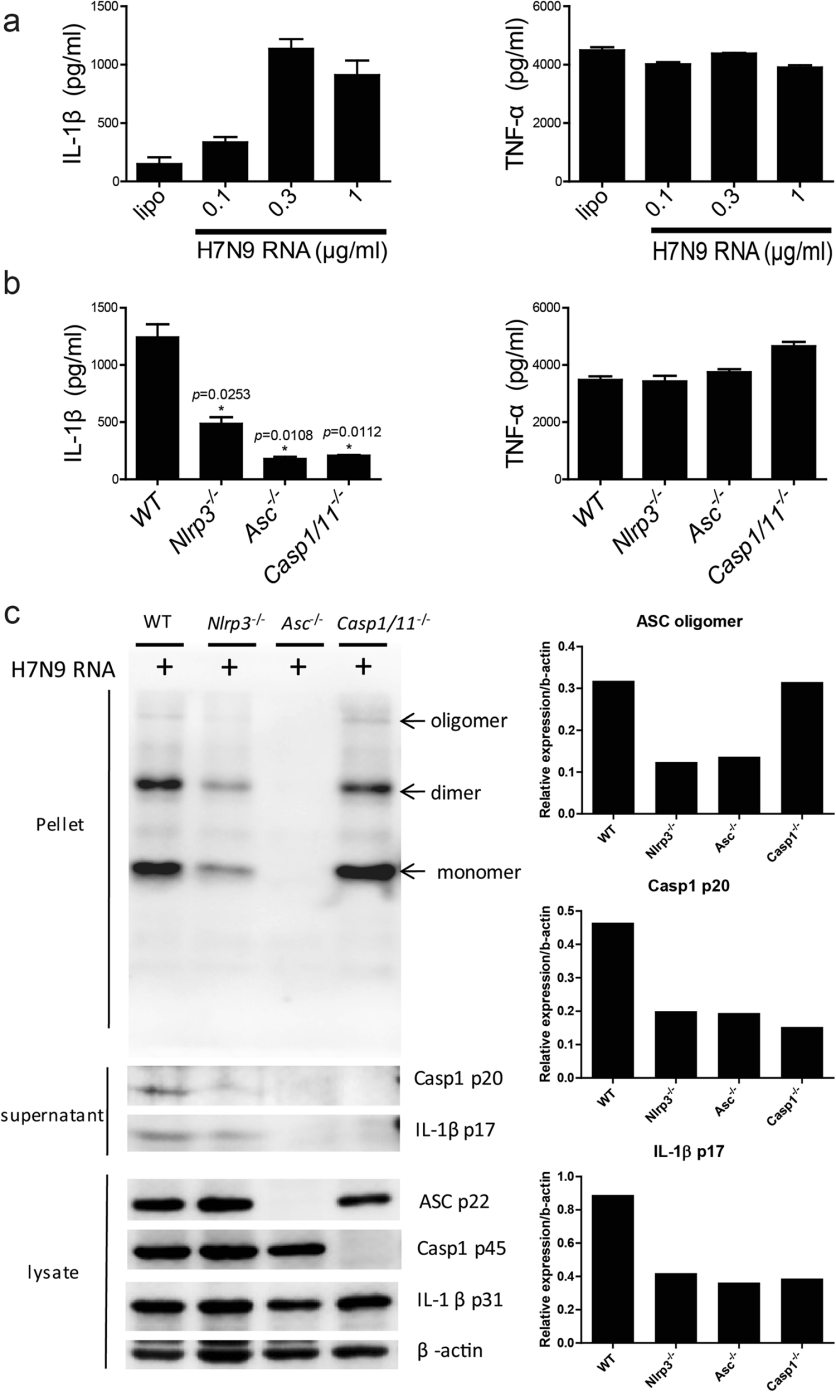
H7N9 RNA activates the NLRP3 inflammasome in BMDCs. (**a**) BMDCs were primed with 500 ng/ml LPS and transfected with different dose of H7N9 RNA for 12 hours, the supernatant were harvest and the levels of IL-1β and TNF-α were measured by ELISA. (**b**) BMDCs from wild type and *Nlrp3* ^−/−^, *Asc* ^−/−^, *Casp1/11* ^−/−^ mice were primed as in (**a**) and transfected with 0.5 ug/ml H7N9 RNA for 12 hours, supernatant were collected for ELISA as indicated. (**c**) Supernatants or cell lysates from H7N9 RNA-stimulated BMDCs were harvested and the expression of proteins was analysed by immunobloting. The upper lanes, marked as pellet, showed the oligomerization of ASC. The right panel indicated the relative density of the immunoblotting. The full-length gels were displayed in Supplementary Figure 3. Values represent the mean of triplicate samples ± SD. Data are representative of three independent experiments. *P < 0.05.

It has been reported that H1N1 RNA plays a critical role in activating the NLRP3 inflammasome^11^. To assess whether the NLRP3 inflammasome could be activated during H7N9 challenge, we transfected LPS-primed BMDCs (bone marrow derived dendritic cells) with H7N9 RNA and measured the production of IL-1β. As expected, robust IL-1β secretion was detected in the H7N9 RNA-transfected samples, while the production of an inflammasome-independent cytokine TNF-α was unaltered (Fig. 1a), suggesting that H7N9 RNA-induced IL-1β production was likely dependent on inflammasome activity. To ensure the role of inflammasome in H7N9 RNA-mediated induction of IL-1β, we generated BMDCs from *Nlrp3* ^−/−^, *Asc* ^−/−^ and *Casp1/11* ^−/−^ mice. Transfection of LPS-primed BMDCs from such mice led to significantly compromised IL-1β secretion compared to wild type controls (Fig. 1b). And western blotting revealed that caspase-1 activation and ASC oligomerization were also decreased in BMDCs from *Nlrp3* ^−/−^ and *Asc* ^−/−^ mice (Fig. 1c), which might account for the compromised IL-1β production. Thus, our results suggest that H7N9 RNA activates caspase-1 via the NLRP3 inflammasome in mouse dendritic cells.

### Deficiency of NLRP3 or caspase-1 protects mice against H7N9 infection associated morbidity and mortality

**Figure 2:**
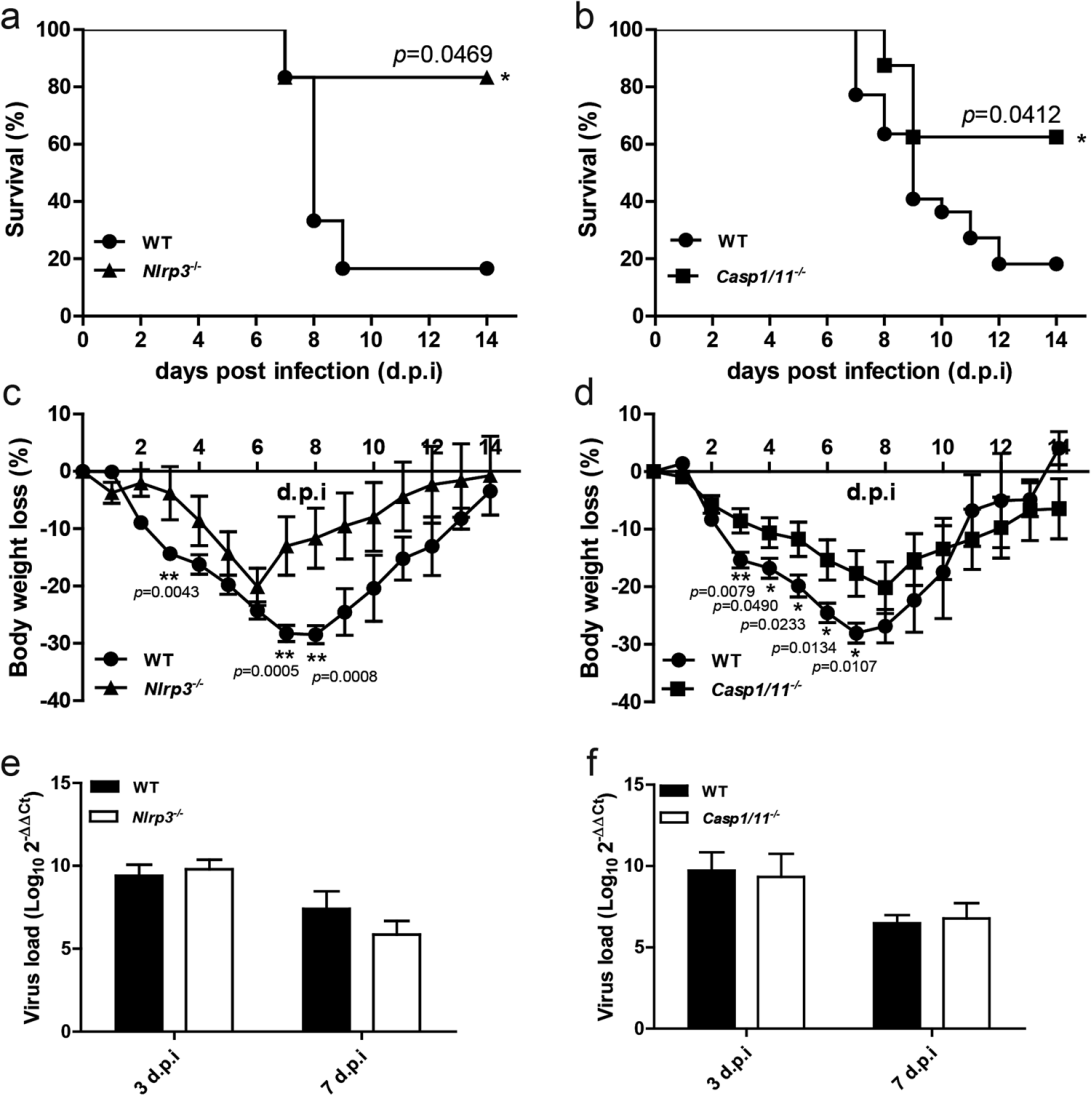
Deficiency of NLRP3 or caspase-1 protects mice against H7N9 infection. (**a**) Wild-type (WT) (n = 18), *Nlrp3* ^−/−^ (n = 18), (**b**) WT (n = 22), C*asp1/11* ^−/−^ (n = 20) mice were infected intranasally with 5 × 10^4^ TCID_50_ of H7N9 virus, and survival was monitored daily for 14 days. *P < 0.05, Log-rank (Mantel-Cox) test. (**c**,**d**) The percentages of weight loss of WT, *Nlrp3* ^−/−^ and *Caspase1/11* ^−/−^ were measured during 14 days. (**e,f**) WT, *Nlrp3* ^−/−^ and *Casp1/11* ^−/−^ mice were infected as in (**a**,**b**) and the lungs were harvested at 3,7 days post infection (d.p.i), each group contained 3 mice, viral load were determined via real time PCR, values represent the mean of triplicate samples ± SD. Data are pooled from three independent infection experiments. **P < 0.01, *P < 0.05.

Next, we investigated the function of NLRP3 inflammasome during H7N9 infection *in vivo*. To this end, age and gender matched WT, *Nlrp3* ^−/−^ and *Casp1/11* ^−/−^ mice were intranasally (i.n.) infected with H7N9 virus as described^15^. We found that the *Nlrp3* ^−/−^ mice survived significantly better than the WT controls (Fig. 2a). Consistently, the mortality of *Casp1/11* ^−/−^ mice was also significantly lower compared to WT controls 14 days post infection (d.p.i) (Fig. 2b). In terms of body weight changes, *Nlrp3* ^−/−^ mice had less weight loss compared to WT controls (P < 0.01 at 3, 7, 8 d.p.i) (Fig. 2c). Moreover, WT mice lost significantly more body weights than *Casp1/11* ^−/−^ mice from 3 to 7 d.p.i (P < 0.01 at 3 d.p.i and P < 0.05 at other time points) (Fig. 2d). However, on 10 d.p.i the survived WT mice began to recover quicker than *Casp1/11* ^−/−^ mice. The gain of body weight started at 7 d.p.i for WT but 8 d.p.i. for *Casp1/11* ^−/−^ mice. By 14 d.p.i, WT mice returned to the initial level of body weight but the *Casp1/11* ^−/−^ mice did not (Fig. 2d). Notably, no significant difference for H7N9 viral quantity in the lungs was observed between the infected *Nlrp3* ^−/−^ or *Casp1/11* ^−/−^ vs. WT mice (Fig. 2e,f).

### Deficiency of NLRP3 or caspase-1 decreases pulmonary inflammation during H7N9 challenge

**Figure 3:**
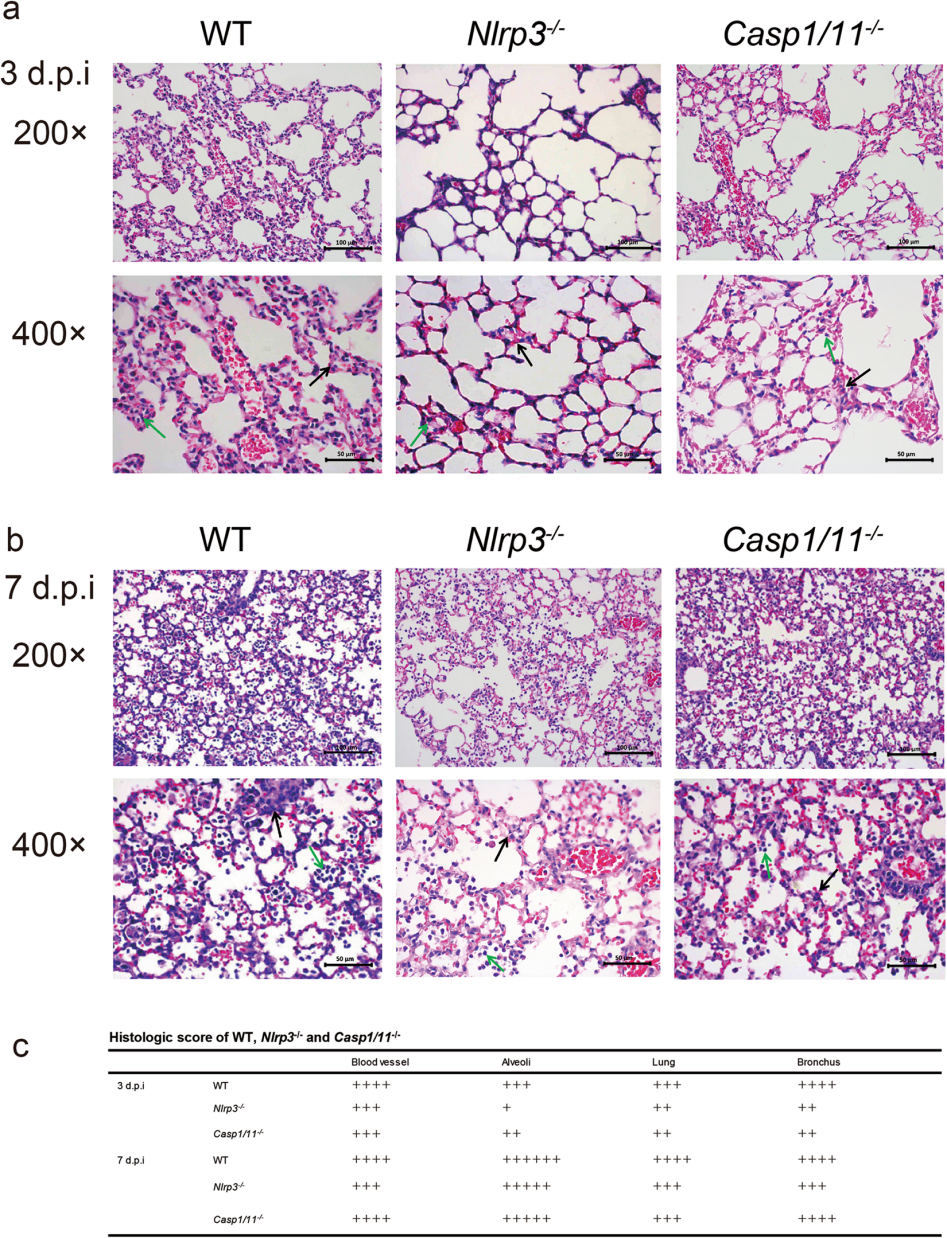
Deficiency of NLRP3 or caspase-1 decreases pulmonary inflammation during H7N9 infection. WT, *Nlrp3* ^−/−^ or *Casp1/11* ^−/−^ mice were infected as in Fig. 2 and the lungs were harvested on 3 (**a**) or 7 (**b**) days post infection (d.p.i), then the tissues were fixed and processed for H&E staining and microscopy. The original magnification of the top panels and the bottom panels are 200× and 400× (with the corresponding scale bar on the images). In the 400× magnification, the black arrows indicate the thickening of alveolar septum and the green arrows show inflammatory cells infiltration. (**c**) For scoring results of pulmonary pathology of WT, *Nlrp3* ^−/−^ and *Casp1/11* ^−/−^ mice, 4 slides from each mouse were monitored. 6 mice representing each genotype were examined at indicated time points. Data are representative of three independent experiments.

To study the pathological changes in the H7N9 infected *Nlrp3* ^−/−^, *Casp1/11* ^−/−^ and WT mice, lung tissues were collected and H&E stained samples were examined by microscopy. On 3 d.p.i, infiltration of inflammatory cells was observed in the infected lungs of all strains of mice. There was an obvious thickening of alveolar septum, destruction of partial alveolar structure, as well as pulmonary septal rupture (Fig. 3a and c). However, infiltration of inflammatory cells to the lungs of WT mice was much more severe than that in *Nlrp3* ^−/−^ or *Casp1/11* ^−/−^ mice, especially in the bronchus. In addition, the destruction of alveolar structure in the lungs of *Nlrp3* ^−/−^ and *Casp1/11* ^−/−^ mice was weaker than that in WT mice (Fig. 3a and c). On 7 d.p.i, all strains of mice showed more severe infiltration of inflammatory cells to the lungs comparing to that on 3 d.p.i (Fig. 3b and c). Nonetheless, the pulmonary pathology of *Nlrp3* ^−/−^ and *Casp1/11* ^−/−^ mice was still milder than that in WT mice on 7 d.p.i (Fig. 3b and c). Collectively, these results suggested a role for NLRP3 and caspase-1 in promoting pathological changes in the lungs of mice infected with H7N9.

### Caspase-1 mediates the recruitment of proinflammatory leukocytes to the lungs during H7N9 infection

**Figure 4:**
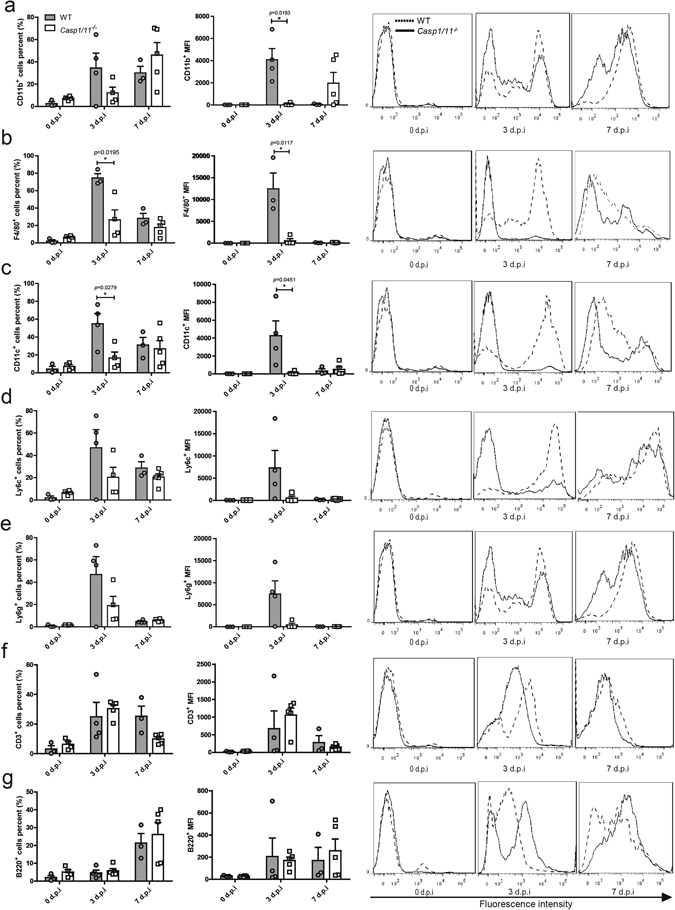
Deficiency of caspase-1 reduces the proinflammatory leukocytes recruitment to the lungs during H7N9 infection. WT and *Casp1/11* ^−/−^ mice were infected as described in the legend for Fig. 2. The BALFs were collected at the indicated time points and leukocytes were stained with specific antibodies before flow cytometric analysis (FACS). Graphs on the left panel represent the mean values from 3–5 mice per group and are expressed as the mean ± SD. *P < 0.05. Histograms on the right panel show representative FACS data. The solid line denotes the *Casp1/11* ^−/−^ mice, and the dashed line denotes the WT mice. Data are pooled from three independent infection experiments. *P < 0.05.

Caspase-1 is the critical executor of inflammasome activation mediating the cleavage of proIL-1β. Therefore, we further studied the impact of caspase-1 deficiency on the infiltration of proinflammatory leukocytes to the lungs after H7N9 infection. The single-cell suspensions, prepared from the bronchoalveolar lavage fluid (BALF) of H7N9 infected WT or *Casp1/11* ^−/−^ mice on 0, 3 and 7 days post infection were stained with anti -CD11b, -F4/80, -CD11c, -Ly6c, -Ly6g, -CD3 and -B220 antibodies conjugated to different fluorescent conjugates, and analyzed through flow cytometry. In both WT and *Casp1/11* ^−/−^ mice, the percentages of leukocytes expressing CD11b, F4/80, CD11c, Ly6c and Ly6g peaked around 3 d.p.i, then declined (Fig. 4a–e). The percentages of F4/80 (74.43% ± 5.027%) and CD11c (54.85% ± 11.50%) positive cells in the WT BALF samples were significantly higher than that in *Casp1/11* ^−/−^ BALF samples (F4/80: 26.48% ± 11.42%; CD11c: 16.52% ± 6.654%) on 3 d.p.i. Meanwhile, the mean fluorescence intensities (MFIs) of CD11b (4,092 ± 1,409), F4/80 (12,450 ± 3,616) and CD11c (4,270 ± 1,642) in WT BALF cells were significantly increased compared with that of *Casp1/11* ^−/−^ mice (CD11b: 80.10 ± 54.18; F4/80: 531.0 ± 483.4; CD11c: 119.7 ± 94.97) on 3 d.p.i (Fig. 4a–c). For Ly6c and Ly6g, albeit there was no statistical difference between the WT and *Casp1/11* ^−/−^ mice, the percentage was at least more than two folds higher in the WT mice than that in *Casp1/11* ^−/−^ mice; and the differences of the MFIs in those two groups were even bigger on 3 d.p.i (Fig. 4d and e). For lymphocytes, CD3 positive cells and B220 positive cells showed no significant differences between WT and *Casp1/11* ^−/−^ mice on both 3 d.p.i and 7 d.p.i (Fig. 4f and g). Thus, deficiency of caspase-1 reduced the proinflammatory leukocytes recruitment to the lungs upon H7N9 IAV challenge.

### Deficiency of caspase-1 or NLRP3 results in attenuated proinflammatory cytokine and chemokine production upon H7N9 infection

**Figure 5:**
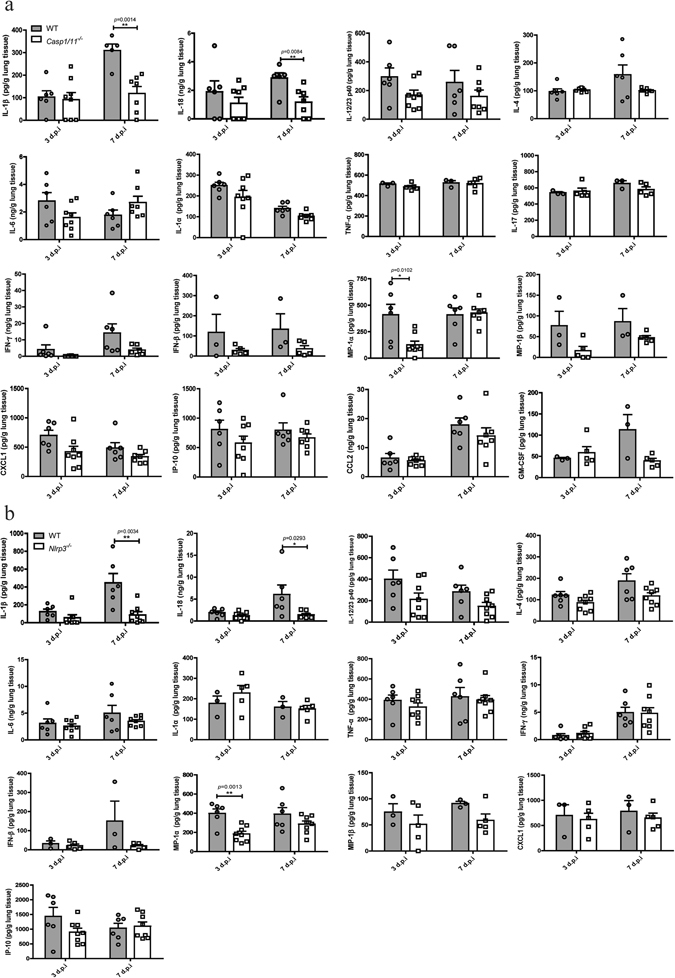
Deficiency of caspase-1 or NLRP3 reduces the production of proinflammatory mediators upon H7N9 infection. WT, *Casp1/11* ^−/−^ (**a**) or *Nlrp3* ^−/−^ (**b**) mice were infected as in Fig. 2 and the lungs were harvested on 3 or 7 d.p.i, then tissue homogenates were taken for determinations of cytokines and chemokines production as indicated. Values represent the mean of samples ± SD. Data are pooled from two independent infection experiments. **P < 0.01, *P < 0.05.

It is reported that the caspase-1, activated by inflammasome, plays a critical role in murine proinflammatory responses to IAV infection^11, 12, 16^. To investigate the role of caspase-1 in H7N9 infection related inflammation, cytokines and chemokines in the supernatants of lung homogenates were analyzed (Fig. 5). In *Casp1/11* ^−/−^ lung tissues, the levels of IL-1β (118.8 ± 30.68 pg/g tissue) and IL-18 (1.168 ± 0.3761 ng/g tissue) were significantly lower than those in WT tissues (IL-1β: 309.5 ± 28.54 pg/g; IL-18: 2.856 ± 0.3617 ng/g, respectively) on 7 d.p.i (P < 0.01) (Fig. 5a). Other cytokines such as IL-12/23p40, IL-1α, IFN-γ and IFN-β also exhibited decreased level in *Casp1/11* ^−/−^ lung tissues compared with WT control on either 3 d.p.i or 7 d.p.i, although the differences were not significant (Fig. 5a). The level of IL-6 fluctuates and was lower in the lungs from *Casp1/11* ^−/−^ mice on 3 d.p.i, but higher on 7 d.p.i compared to WT counterparts, without significant differences in both cases (Fig. 5a). Other cytokines such as IL-4, TNF-α and IL-17 did not show significant differences between tissues from *Casp1/11* ^−/−^ and WT mice (Fig. 5a). As for the detected chemokines, MIP-1α was significantly lower in *Casp1/11* ^−/−^ lung tissues than that in WT control on 3 d.p.i (P < 0.05). The levels of other chemokines, including MIP-1β, IP-10, CXCL1, CCL2 and GM-CSF were just slightly lower in the lungs of *Casp1/11* ^−/−^ mice than that in WT counterparts on either 3 d.p.i or 7 d.p.i. Therefore, caspase-1 was involved for an optimal production of IL-1β, IL-18 as well as MIP-1α in the lung in response to H7N9 challenge. To note, the production of above mentioned cytokines and chemokines from *Nlrp3* ^−/−^ mice showed a similar pattern as that from *Casp1/11* ^−/−^ mice (Fig. 5b), indicating that deficiency of NLRP3 inflammasome genes reduced the secretion of several critical proinflammatory mediators in the lung of mice upon H7N9 IAV challenge. In addition, the production of these cytokines and chemokines in the serum of *Nlrp3* ^−/−^, *Casp1/11* ^−/−^ and WT mice showed a similar trend with that from the lung homogenates (Supplementary Fig. S1).

### ASC and IL-1 receptor mediated signal promoted mortality and weight loss of mice after H7N9 infection

**Figure 6:**
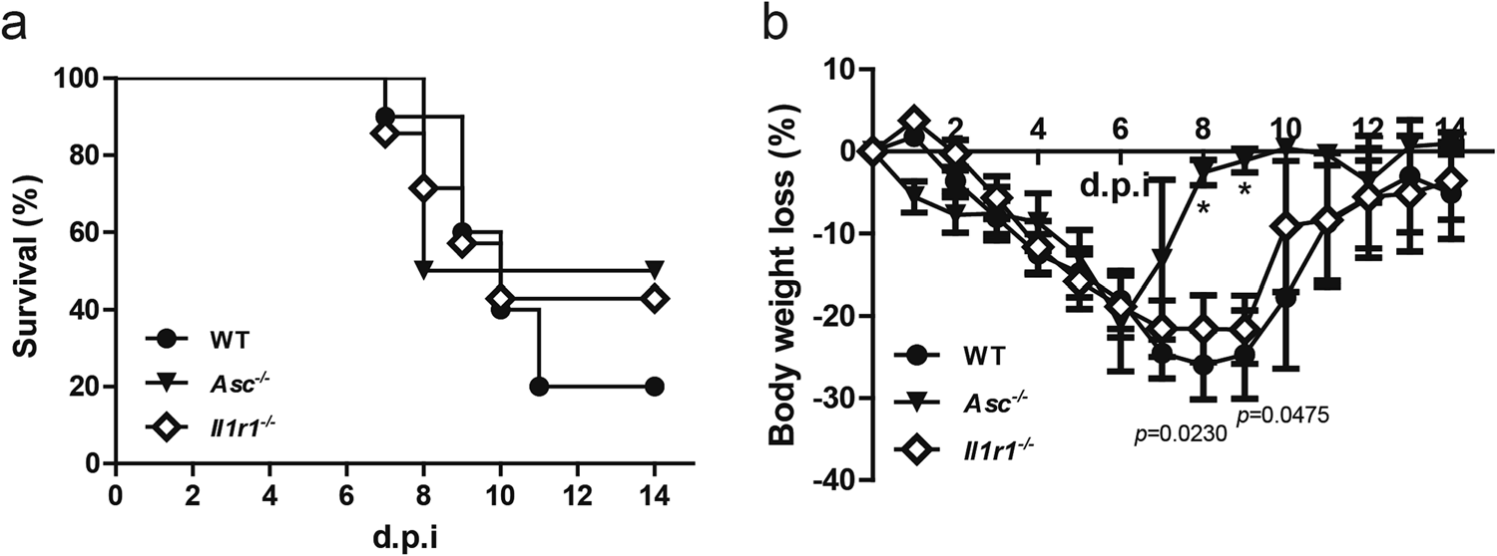
Deficiency of ASC or IL-1 receptor reduces the mortality and weight loss after H7N9 infection. (**a**) WT (n = 18), *Asc* ^−/−^ (n = 16) and *Il1r1* ^−/−^ (n = 14) mice were infected as in Fig. 2, and survival was monitored daily for 14 days. (**b**) The percentage of weight loss from mice shown in (**a**). The weight loss between WT and *Asc* ^−/−^ mice showed statistic significance (P < 0.05) at 8 and 9 d.p.i. Data are pooled from three independent infection experiments. *P < 0.05.

The adaptor protein ASC plays a vital role in the NLRP3 inflammasome formation, which is required for caspase-1 activation and maturation of IL-1β. The mature IL-1β binds to IL-1 receptor 1, the functional receptor of IL-1β, induces downstream signal transduction and executes inflammatory responses. Thus, we set out to determine whether ASC and IL-1R1 play a role similar as that of NLRP3 and caspase-1 during H7N9 IAV infection. To this end, we infected *Asc* ^−/−^ and *Il1r1* ^−/−^ mice with H7N9 virus and monitored their weight change and mortality daily. Our results demonstrated that the mortality of *Asc* ^−/−^ and *Il1r1* ^−/−^ mice was lower compared with that of the WT mice (Fig. 6a). The WT mice also suffered more weight loss than these knock-out animals (from 7 to 10 d.p.i) after H7N9 challenge. Of note, the difference for body weight loss between WT and *Asc* ^−/−^ mice was statistically significant (P < 0.05) on 8 and 9 d.p.i. (Fig. 6b).

### Deficiency of ASC or IL-1 receptor decreases pulmonary inflammation upon H7N9 infection

**Figure 7:**
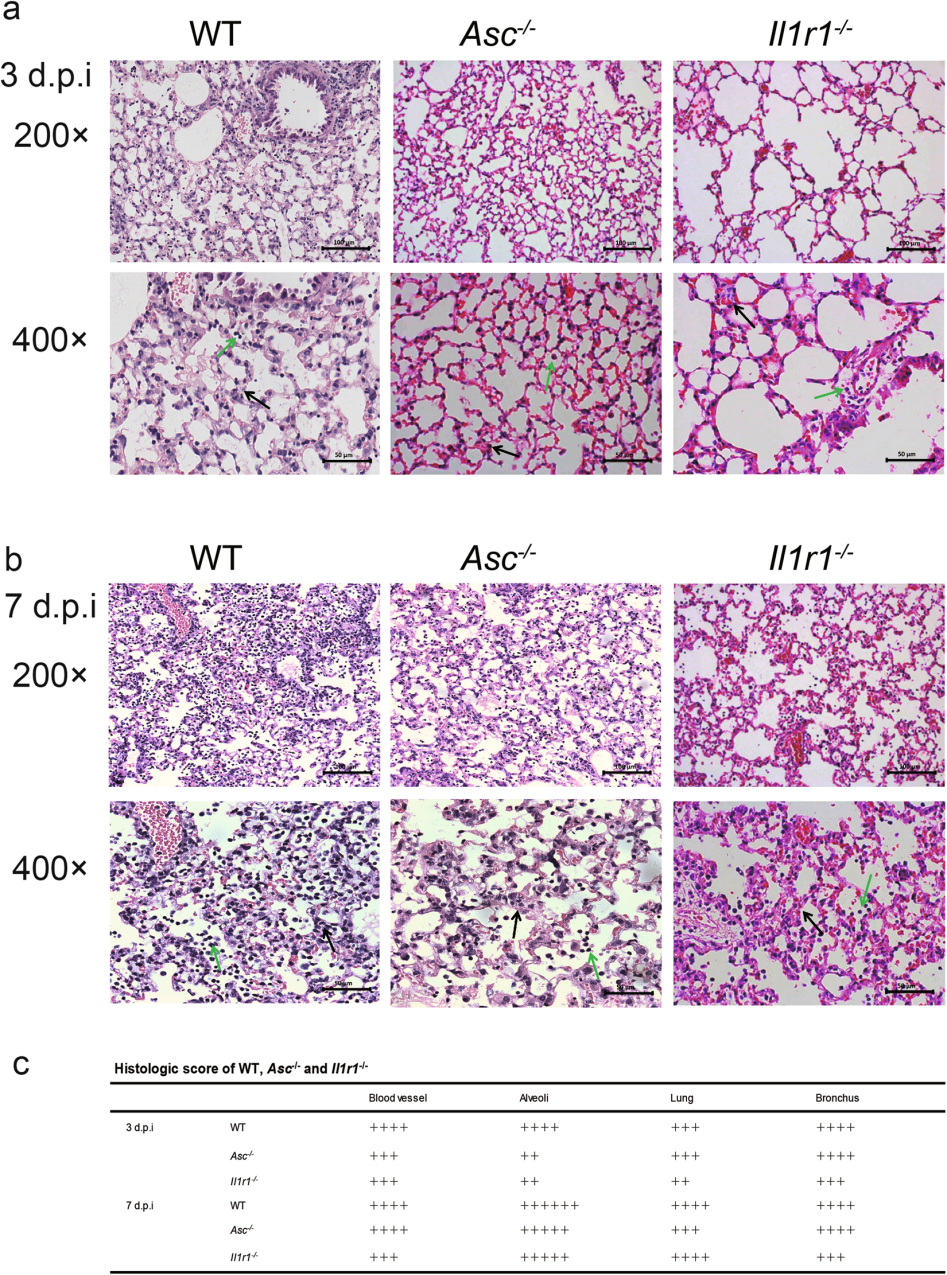
Deficiency of the ASC or IL-1 receptor decreases pulmonary inflammation upon H7N9 infection. (**a,b**) WT or *Asc* ^−/−^, *Il1r1* ^−/−^ mice were infected as in Fig. 2 and the lungs were harvested on 3d.p.i or 7d.p.i, then the tissues were fixed and processed for H&E staining and microscopic analyses. The original magnification of the top panels and the bottom panels are 200× and 400× (with the corresponding scale bar on the images). In the 400× magnification, the black arrows indicate the thickening of alveolar septum and the green arrows show inflammatory cells infiltration. (**c**) For scoring results of pulmonary pathology of WT, *Asc* ^−/−^ and *Il1r1* ^−/−^ mice, 4 slides from each mouse were monitored. 4 mice representing each genotype at specific time points were used for experiments. Data are representative of three independent infection experiments.

To analyze the pulmonary inflammation of *Asc* ^−/−^ and *Il1r1* ^−/−^ mice, lung tissues of these mice were harvested for H&E staining and microscopy. On 3 d.p.i, more inflammatory cells were recruited to the alveoli and blood vessels in the lungs of WT mice compared with that of the *Asc* ^−/−^ or *Il1r1* ^−/−^ mice, and more inflammatory cells infiltrated into the bronchus resulting in a local blockade (Fig. 7a and c). Moreover, the pulmonary exudation was even milder in the *Il1r1* ^−/−^ mice compared with WT mice (Fig. 7a and c). On 7 d.p.i, all mice suffered more severe inflammation than that observed on 3 d.p.i. Furthermore, there were more inflammatory cells infiltrated in the alveoli of WT mice than in *Asc* ^−/−^ or *Il1r1* ^−/−^ mice (Fig. 7b and c). Moreover, the damage in the lung of *Asc* ^−/−^ mice was milder compared with that in WT mice, and there was less inflammatory exudation in *Il1r1* ^−/−^ mice than in WT controls (Fig. 7b and c). Therefore, our results suggest that signals mediated by ASC and IL-1 receptor lead to more severe disease upon H7N9 IAV challenge.

## Discussion

Aberrant and excessive inflammation during H7N9 viral infection is linked to severe morbidity and mortality in human patients^17^. A recent study demonstrated the histological distribution of inflammation-related genes expression in the lungs of BALB/c mice infected with H7N9 influenza A virus^18^. Significantly higher expression levels of NLRP3, RIP3, IL-1β and TNF-α in the lung of H7N9-infected mice were noted, indicating possible role of these molecules in driving lung pathogenesis^18^. For example, the involvement of TNF-α activated signaling pathway during influenza virus infection was clearly demonstrated^19, 20^. However, the exact roles of other molecules involved in inflammatory response during H7N9 challenged remained elusive. Specifically, the role of inflammasome signaling had not been defined before our present work.

Here, using the gene knockout mice we demonstrated that activation of the NLRP3 inflammasome was detrimental to the host upon H7N9 IAV infection. We noted that WT mice lost more body weight than *Casp1/11* ^−/−^ *, Asc* ^−/−^ and *Nlrp3* ^−/−^ mice and their overall survival rate was lower during H7N9 infection (Figs 2 and 6). Pathology analysis showed that there were inflammatory responses in the infected lungs of all strains of mice, both at 3 d.p.i and at 7 d.p.i; and the pulmonary inflammations at 7 d.p.i. were more severe than that at 3 d.p.i.; moreover, the pulmonary inflammations of the WT mice were stronger than *Casp1/11* ^−/−^ *, Asc* ^−/−^ and *Nlrp3* ^−/−^ mice (Figs 3 and 7). Flow cytometric analysis showed that inflammatory cells has started to infiltrate into the alveolar spaces in the BALF at 3 d.p.i. It seemed that CD11b^+^ or F4/80^+^ (high-expressed on microphages) or CD11c^+^ (high-expressed on dendritic cells) cells in the BALF of WT mice were significantly more than those in *Casp1/11* ^−/−^ mice at 3 d.p.i. There were also Ly6c^+^ (expressed on monocytes), Ly6g^+^ (high-expressed on neutrophils) and CD3^+^ (expressed on T cells) in the BALF of WT mice exhibited higher level, though it was not significant differences (Fig. 4). However, except the CD11b^+^ cells were retained and B220+ (expressed on B lymphocytes) cells were increased, the percentage of the most infiltrated cells were decreased in the BALF at 7 d.p.i. (Fig. 4), which seem to be “contrast” to the severer pulmonary inflammation pathology observation at 7 d.p.i. (Figs 3 and 7).

Nevertheless, the rationale may lie in the sequential process of H7N9 virus infection in the WT mice. The H7N9 virus was intranasally inoculated and inhaled into bronchoalveolar spaces, then infected the epithelial cells. At the early stage of first few rounds of viral replication (24~72 hours), the local inflammation was limited (Fig. 3a), and macrophages/dendritic cells and a few other cells started to be recruited to the infected area by chemokine MIP-1α (Figs 4a–c and 5a). The production of IL-1β was still at low level at this stage (Fig. 5a). With the development of disease, at 7 d.p.i., severe inflammation occurred in the infected lung tissue (Fig. 3b). With the migration of the lymphocytes, especially B cells, to the inflammatory area (Fig. 4g), the total number of infiltrated inflammatory cells were increased but the percentage of monocytes, macrophages, dendritic cells were decreased (Supplementary Fig. S2, Fig. 4). The infiltrated inflammatory cells produced large amount of IL-1β (Fig. 5), which accelerated the pulmonary inflammation.

From 7 d.p.i. to 14 d.p.i, the body weight of survived WT mice recovered completely, whereas *Casp1/11* ^−/−^ mice could not returned to the initial level (0 d.p.i) (Fig. 2d). Microscopic analyses revealed that on 14 d.p.i, most tissues from WT mice exhibited complete recovery, while the *Casp1/11* ^−/−^ tissues still manifested strong inflammation (data not shown). Thus, WT mice suffered more due to the lung damage and inflammation in the early phase (before 7 d.p.i) of H7N9 infection and either died of organ failure, or recovered at the later phase. In contrast, *Casp1/11* ^−/−^mice did not develop as strong inflammation and lung damage as seen in WT counterparts in the early phase, consequently fewer of them died. However, *Casp1/11* ^−/−^ mice failed to recover completely at the later phase. These results might suggest a potentially dual role for caspase-1 in H7N9 infection, detrimental role to the host upon H7N9 infection before 7 d.p.i. and possibly necessary role for recovery from the lung inflammation after 7 d.p.i. phase, likely due to a contribution to tissue regeneration. But it should be kept in mind that the comparison between 20% surviving WT and 60% surviving *Casp1/11* ^−/−^ is not justified, and more stringent experiments are needed for further explorations on the role of caspase-1 to host recovery.

Studies on the role of NLRP3-caspase-1 inflammasome during IAV infection produced conflicting results. Some studies have demonstrated that NLRP3-caspase-1 inflammasome is protective against H1N1 infection through viral clearance or virus induced pulmonary necrosis^11, 12^_._ Inflammation is essential to protect host from infection, but can also cause tissue damage or dysfunction of important organs and lead to death^21^. Dysregulation of the proinflammatory response may result in a “cytokine storm” that contributes to a severe viral pneumonia and serious complications in the lung^21^. The highly pathogenic avian influenza virus H5N1 causes hypercytokinemia and severe tissue damage^22^. It is reported that production of proinflammatory cytokines and chemokines, including IL-6 and IP-10, is remarkably increased in H7N9 infected patients^17, 23^, and H7N9 infection associated inflammation in the lungs resulted in rapidly progressive pneumonia and development of ARDS in the majority of hospitalized patients^24, 25^. In addition, the H5N1 IAV infected patients who progressed severe lung injury have elevated levels of CCL2, IP-10, CXCL9 and IL-8 compared with the seasonal H1N1 infected patients^26^. Therefore, for the highly pathogenic influenza virus the hyperactivated proinflammatory responses may be detrimental for the host. Moreover, a recent study demonstrates that NLRP3 inflammasome plays both a protective and detrimental role during H1N1 and H3N2 infection of mice depending on the disease phase^13^. In our study, we found the inflammasome activation was deleterious in the case of H7N9 infection, possibly due to overwhelming and fatal acute inflammation in the lungs following H7N9 infection. Pathology and infiltrating cellular analyses as well as cytokine and chemokine analyses all supported this possibility (Figs 3–5). Pathology results revealed that on 3 d.p.i, there were more inflammatory cells around the blood vessels and bronchus in WT mice compared with *Casp1/11* ^−/−^ mice (Fig. 3). The flow cytometric analyses further demonstrated the abundance of inflammatory cells in the BALF of WT mice compared to *Casp1/11* ^−/−^ counterparts (Fig. 4), which were consistent with the observed pulmonary pathological changes.

Both *Casp1/11* ^−/−^ and *Nlrp3* ^−/−^ mice produced relatively less level of IFN-β in the lung compared with control mice. Although no significant difference was noted, these data indicated a possible role for the NLRP3-caspase-1 inflammasome signaling in affecting the type I interferon production. Theoretically, type I interferon response plays a role in limiting viral replication, however, we did not observe any significant difference in viral load between *Casp1/11* ^−/−^ or *Nlrp3* ^−/−^ versus WT mice. It is likely that the H7N9 could employ strategies to counteract the generation and function of type I interferon.

Chemokines are the driving factors promoting the recruitment of inflammatory cells. MIP-1α (CCL3) can activate granulocytes and lead to acute neutrophilic inflammation. It also induces the synthesis and release of IL-1β, IL-6 and TNF-α from fibroblasts and macrophages^27^. MIP-1α level is increased in H7N9 and H5N1 infected mice, but not in H9N2 infected animals^28^. We also noticed a clear increase of MIP-1α in WT mice upon H7N9 infection (Fig. 5). Interestingly, this elevation was abrogated in the early phase (3 d.p.i) in the absence of caspase-1 (Fig. 5). We reasoned that the MIP-1α production may be, directly or indirectly, influenced by NLRP3 or caspase-1-dependent IL-1β and/or IL-18 secretion.

IL-1β showed significant reductions in *Nlrp3* ^−/−^ and *Casp1/11* ^−/−^ mice compared with WT on 7 d.p.i (Fig. 5). IL-1β is a critical proinflammatory cytokine that is increased during infections with H1N1 and H5N1 influenza viruses^29^. IL-1β also mediates the recruitment of monocytes and neutrophils into the lung^30^. Deficiency of IL-1β or its receptors manifests different responses to respiratory challenge with influenza virus^31–33^. Hence, the function of IL-1β in controlling IAV infections remains obscure. Our data showed that *Il1r1* ^−/−^ mice exhibited attenuated tissue pathogenesis upon H7N9 infection (Figs 6 and 7).

During the preparation of this manuscript, we noticed that another group found the PB1-F2 protein derived from H7N9 virus also activates the NLRP3 inflammasome^34^. Probably, the viral particle of H7N9 contains multiple factors such as RNA and PB1-F2 that can be detected by the host inflammasome. Beside NLRP3 inflammasome, whether other inflammasomes, such as NLRP1, IPAF, AIM2, could also be involved in H7N9 infection needs to be investigated in further study.

In summary, our present work demonstrates that the NLRP3-caspase-1 inflammasome is deleterious for the mammalian host during H7N9 influenza virus infection. Therefore, fine-tuning the NLRP3-caspase-1 activity or IL-1-mediated signaling should be beneficial to control H7N9-associated lethal pathogenesis.

## Methods

### Virus

A/Shanghai/4664 T/2013(H7N9) (GenBank No. KC853225-KC853232) strain was used in this study. Isolation of H7N9 virus was described before and the viral titer was measured via tissue culture infective dose (TCID_50_) assay^15, 25^. All experiments related to H7N9 virus were performed in biosafety level 3 laboratories in Shanghai Public Health Clinical Center (SPHCC), and followed the standard operating protocols approved by the Institutional Biosafety Committee at SPHCC, Fudan University.

### Mice

All mice used in our experiments are on a C57BL/6 genetic background and all experiments were carried out with age and gender matched mice (8–12 weeks old, female). C57BL/6 mice were purchased from the B&K Universal Group Limited (Shanghai, China). *Nlrp3* deficient mice (a gift from Dr. Warren Strober) and *Asc* deficient mice (a gift from Dr. Vishva M. Dixit) had been described^35, 36^. *Caspase-1/11* and IL-1 receptor 1 deficient mice were purchased from the Jackson Laboratory (Bar Harbor, ME, USA). Herein, these mice were described as *Nlrp3* ^−/−^ *, Asc* ^−/−^ *, Casp1/11* ^−/−^ and *Il1r1* ^−/−^. All mice were housed under specific pathogen-free (SPF) condition at the animal facility at Institut Pasteur of Shanghai. Mice were transferred to the Animal Biosafety Level 3 (ABSL3) Laboratory at SPHCC three days before infection. Mice care and experimental procedures complied with national guidelines and were approved by the Institutional Animal Care and Use Committee (IACUC) at SPHCC, Fudan University.

### Cells

Bone marrow derived dendritic cells (BMDCs) were generated as described^37^. Briefly, bone marrow cells were harvest and lysed with RBC lysis buffer, and seeded in 10 cm dish with 10 ml RPMI1640 with GM-CSF (20 ng/ml). The media were refreshed 3 days later and the cells were harvested for experiments on day 6.

### Antibodies

Antibodies for immunoblotting include: rabbit anti-mouse ASC (sc-22514-R; Santa Cruz, CA, USA), mouse anti-mouse caspase-1 (AG-20B-0042; Adipogen, San Diego, CA, USA), rabbit anti-mouse mature and pro–IL-1β (sc-7884; Santa-Cruz, CA, USA) and mouse anti-mouse β-actin (sc-47778; Santa-Cruz, CA, USA).

### H7N9 RNA extraction and transfection into BMDCs

H7N9 RNA was extracted with QIAamp Viral RNA Mini Kit(Qiagen, Hilden, Germany) according to the manufacture’s protocol. And we measured the concentration of viral RNA with Nanodrop 2000 (Thermo Scientific, Madrid, Spain). BMDCs were primed with 500 ng/ml LPS (Sigma-Aldrich, St.Louis, MO, USA) for 4 hours, then the different doses of H7N9 viral RNA were transfected with Lipofectamine 2000 (Invitrogen, Carlsbad, CA, USA) according to the manufacture’s protocol. Briefly, Lipofectamine 2000 (2 ul/ml) and viral RNA were diluted in opti-MEM (Invitrogen, Carlsbad, CA, USA), then mixed together for 20 minutes and the mixture was added into cells. 12 hours later, the supernatant was harvested for ELISA or inflammasome activation assay, and the cells were collected for Western Blotting and ASC oligomerization detection.

### ASC oligomerization detection

ASC oligomer was prepared as described^36^. Brifely, H7N9 RNA transfected BMDCs were lysed with lysis buffer [50 mmol/L Tris (pH 7.5), 150 mmol/L NaCl, 1% Nonidet P-40], pelleted and washed with PBS, and cross-linked with disuccinimidylsuberate (DSS) before subjected to immunobloting.

### Mice infection

Mice were infected with 5 × 10^4^ TCID_50_ of H7N9 virus in a volume of 50 µl intranasally (i.n.) after sevoflurane (Hearem, Shanghai, China) inhalation anaesthesia, and monitored for clinical signs, body weight changes and physical survival during the 14 days observation period. The infected mice were sacrificed on 3, 7 or 14 days post infection for collection of samples for analysis as reported^15^. To monitor the body weight changes and survival rate, 14 to 22 mice were used in each group (n = 14~22). For the rest analysis of murine samples, more than 3 mice were used in each group.

### Analysis of cytokines and chemokines

Lung homogenate supernatants and serum were collected and stored at −80 °C before analysis. The levels of cytokines and chemokines in the collected samples were determined quantitatively using a ProcartaPlex Multiplex Immunoassay kit (eBioscience, Viennna, Australia) according to the manufacturer’s protocol.

### Flow cytometry

Bronchoalveolar lavage fluid (BALF) was obtained via flushing the lungs three times with 0.5 mL/time of PBS. The single-cell suspensions from BALF of H7N9 infected mice were first treated with anti-mouse CD16/CD32 antibody (eBioscience, Vienna, Australia) to block Fc receptor, then surface stained with antibodies against B220, CD3, CD11b, CD11c, Ly6c, Ly6g and F4/80, and fixed with 4% paraformaldehyde before analysis with FACS Array (BD Biosciences, Franklin Lakes, NJ, USA). The final graphical output was generated through FlowJo software (Tree Star, Inc., Ashland, OR, USA).

### Extraction of total RNA and RT-PCR quantification

Total RNA was extracted from lung homogenates using a kit (Qiagen, Hilden, Germany) according to the manufacturer’s protocol. Relative Quantification of H7N9 viral load via RT-PCR was conducted as reported previously^15^. Calculation was carried out by comparing H7N9 Hemagglutinin with GAPDH, which served as an internal control GAPDH (ΔΔCT method).

### Pathology analysis

Excised mouse lung tissues were fixed in 4% paraformaldehyde followed by paraffin embedding and sectioning, then subjected to hematoxylin and eosin (H&E) staining. Images were collected by Leica DMI3000 microscopy with indicated magnifications.

### Histology scoring

Four indexes were analyzed for scoring on each slide: 1) Blood vessel region inflammation, 2) Pulmonary alveoli inflammation, 3) Lung tissue inflammation, 4) Bronchial inflammation. The detailed criteria were listed in Table 1, which was referred to a previous report^38^ and modified by the pathologist, Dr. Y Feng, in author list of this manuscript.

**Table 1:** Histology Scoring Criteria.

Inflammation in different sites
**Blood vessel region inflammation**
++ Some blood vessels are cuffed by inflammatory cells ++++ Most blood vessels are surrounded by a thin layer (1–5 cells thick) of inflammatory cells ++++++ Most blood vessels are surrounded by a thick layer (>5 cells thick) of inflammatory cells
**Pulmonary alveoli inflammation**
++ Increased numbers of inflammatory cells in alveolar walls are evident ++++ As above, plus one to three foci per section showing alveolar exudate and atelectasis ++++++ As above, plus more than three foci per section showing alveolar exudate and atelectasis
**Lung tissue inflammation**
++ Some blood vessels are cuffed by inflammatory cells ++++ A part of lung tissue shows consolidation and interstitial fibrous tissue proliferation ++++++ Consolidation appears in most areas of the lung
**Bronchial inflammation**
++ Some bronchus are cuffed by inflammatory cells ++++ Some bronchus exhibit squamous metaplasia and are blocked by inflammatory exudates ++++++ Most bronchus exhibit squamous metaplasia and are blocked by inflammatory exudates

The three levels of inflammatory response for each index, which was designated as ++, ++++ and ++++++, according to the criteria listed in the above table. The milder inflammation was designated with reduced number of “+” as indicated in respective Figures. To warrant the objectivity of the histology scoring, two pathologists from different labs read each slide in a double-blinded way and an average value was taken as the final result.

### Data analysis

All statistical analysis was conducted using GraphPad Prism Software via parametric unpaired t-test if not noted otherwise (Version 5.0; GraphPad, La Jolla, CA, USA). P < 0.05 was defined as statistically significant.

## Electronic supplementary material

supplementary figures 1, 2 and 3
