# Supplementary material for: The H7N9 influenza A virus infection results in lethal inflammation in the mammalian host via the NLRP3-caspase-1 inflammasome
Source: Sci Rep. 2017 Aug 8;7:7625. doi: 10.1038/s41598-017-07384-5 (PMC5548739; doi:10.1038/s41598-017-07384-5)
Supplement: Supplementary file 1 — supplementary figures 1, 2 and 3 [file 41598_2017_7384_MOESM1_ESM.pdf]

Supplementary Figure

The H7N9 influenza A virus infection results in lethal inflammation in the mammalian host via the NLRP3-caspase-1 inflammasome

Running title: NLRP3 inflammasome mediates deleterious responses to H7N9

Rongrong Ren[1,+], Shuxian Wu[2,+], Jialin Cai[4,+], Yuqin Yang[5,+], Xiaonan Ren[1], Yanling Feng[1], Lixiang Chen[1], Boyin Qin[1], Chunhua Xu[1], Hua Yang[1], Zhigang Song[1], Di Tian[1], Yunwen Hu[1,3], Xiaohui Zhou[1,3,*], Guangxun Meng[2,*]

Supplementary Figures: 3(Fig. S1, Fig. S2, Fig. S3)

Supplementary Figure S1

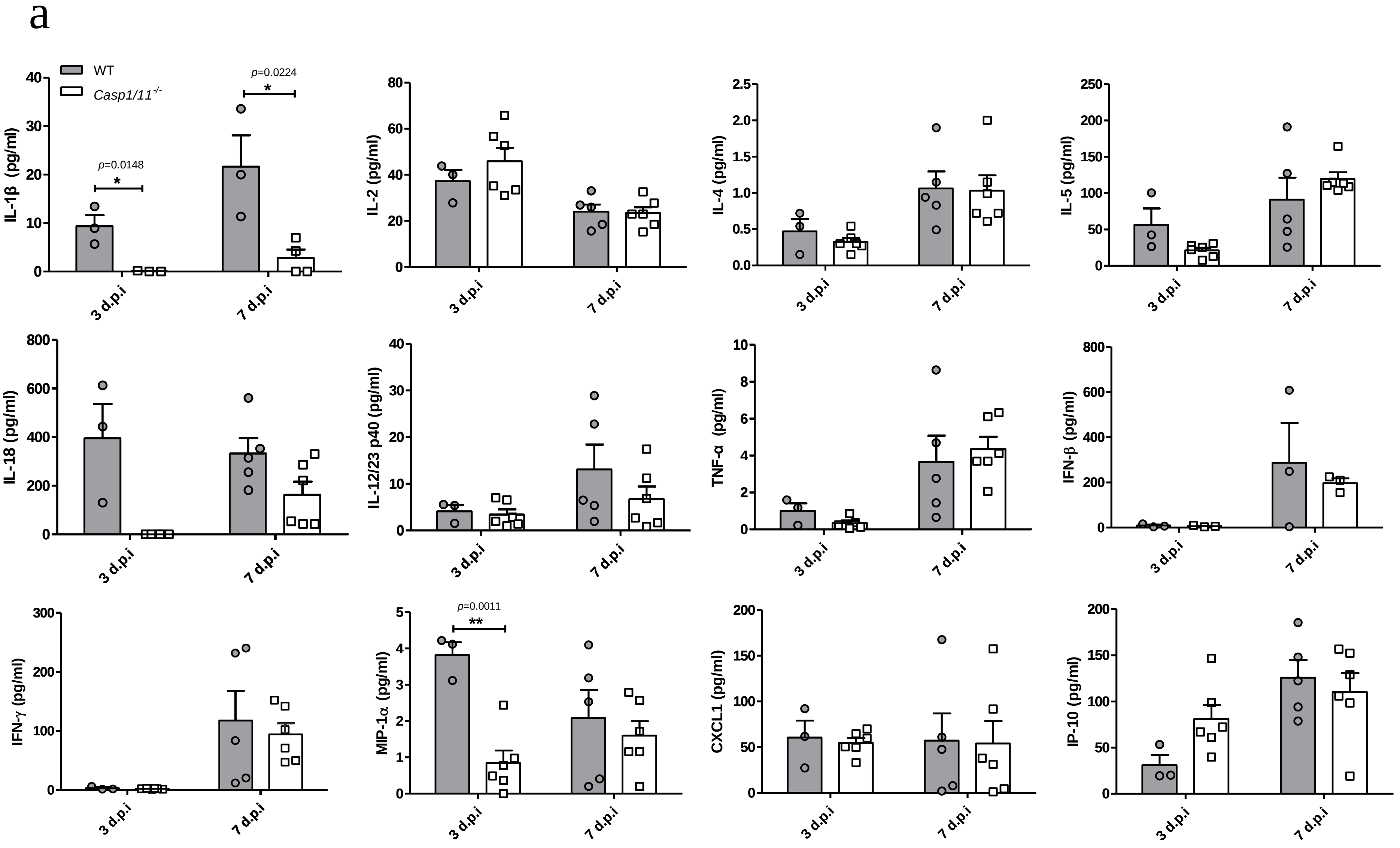

b

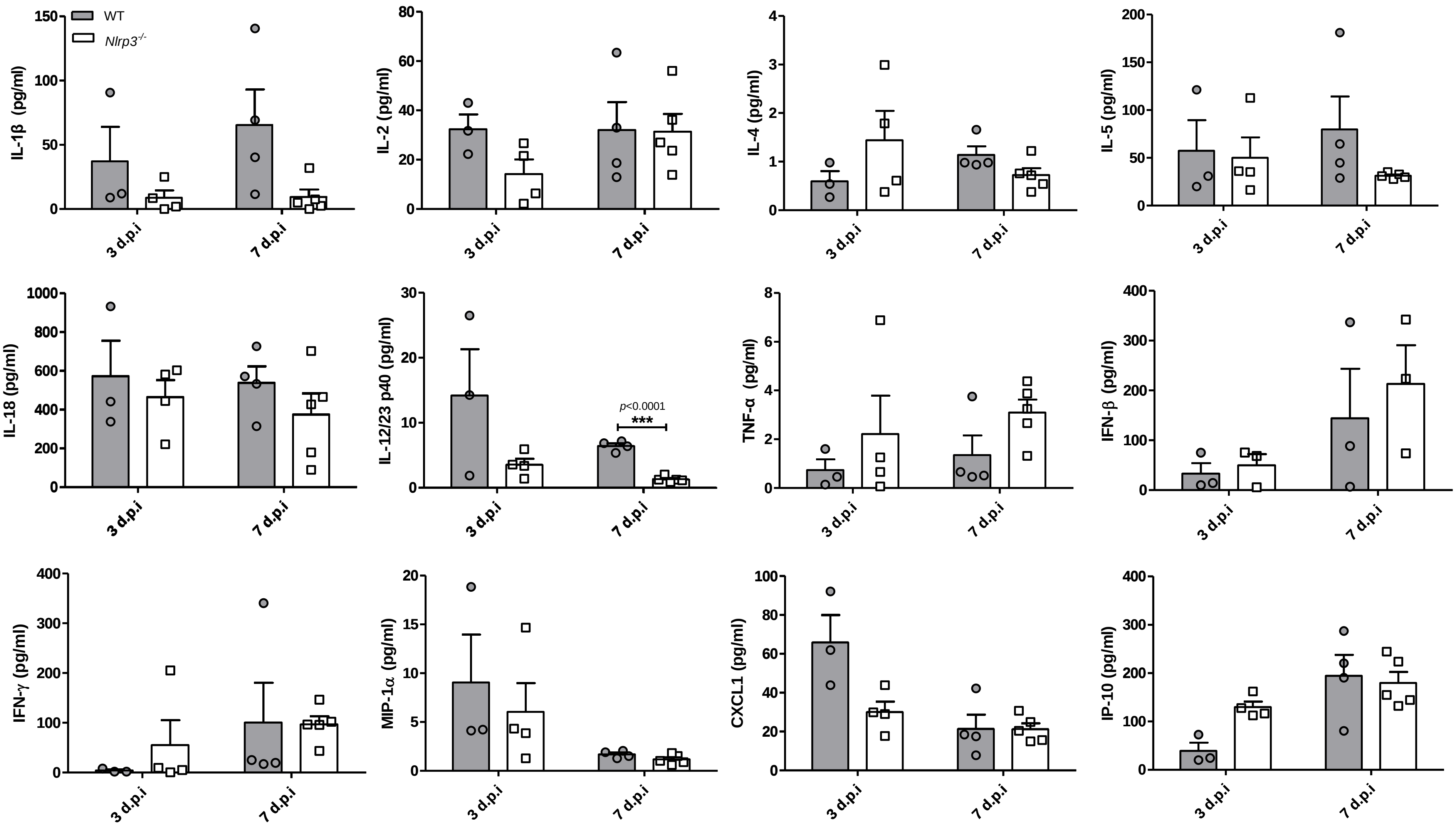

Fig. S1. Deficiency of Nlrp3 or caspase-1 alters the levels of proinflammatory mediators in serum upon H7N9 infection. WT, *Nlrp3*$^{-/-}$ or *Casp1/11*$^{-/-}$ mice were infected intranasally with 5×10^4 $TCID_{50}$ of H7N9 virus and the serum was assessed on 3 or 7 d.p.i. Values represent the mean of samples ± SD. * $P<0.05$, ** $P<0.01$, *** $P<0.0001$ ($n\geq3$).

Supplementary Figure S2

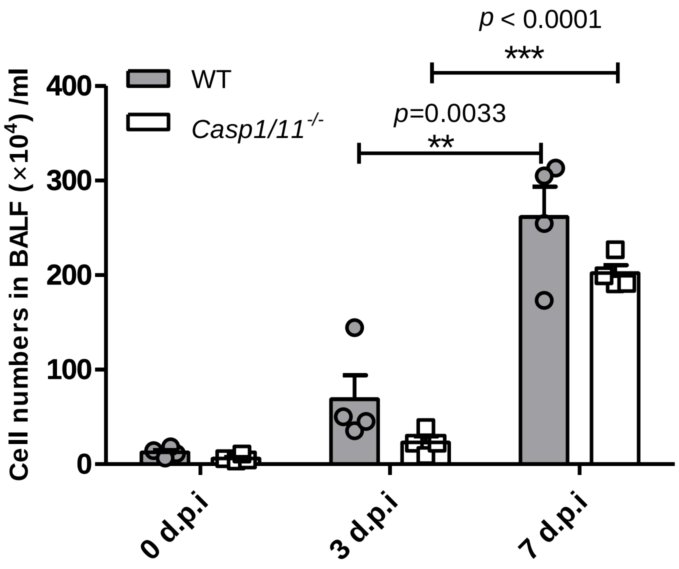

Fig. S2. Number of infiltrated cells in the BALFs of WT and *Casp1/11$^{-/-}$* mice.

WT and *Casp1/11$^{-/-}$* mice were infected intranasally with 5×10^4 $TCID_{50}$ of H7N9 virus. The BALFs were collected at the indicated time points. After light centrifugation, total cell numbers were determined using a cell counting chamber. Values represent the mean of samples $\pm$ SD. Data are representative of three independent infection experiments. **P<0.01, ***P<0.0001 (n=4).

Supplementary Figure S3

a

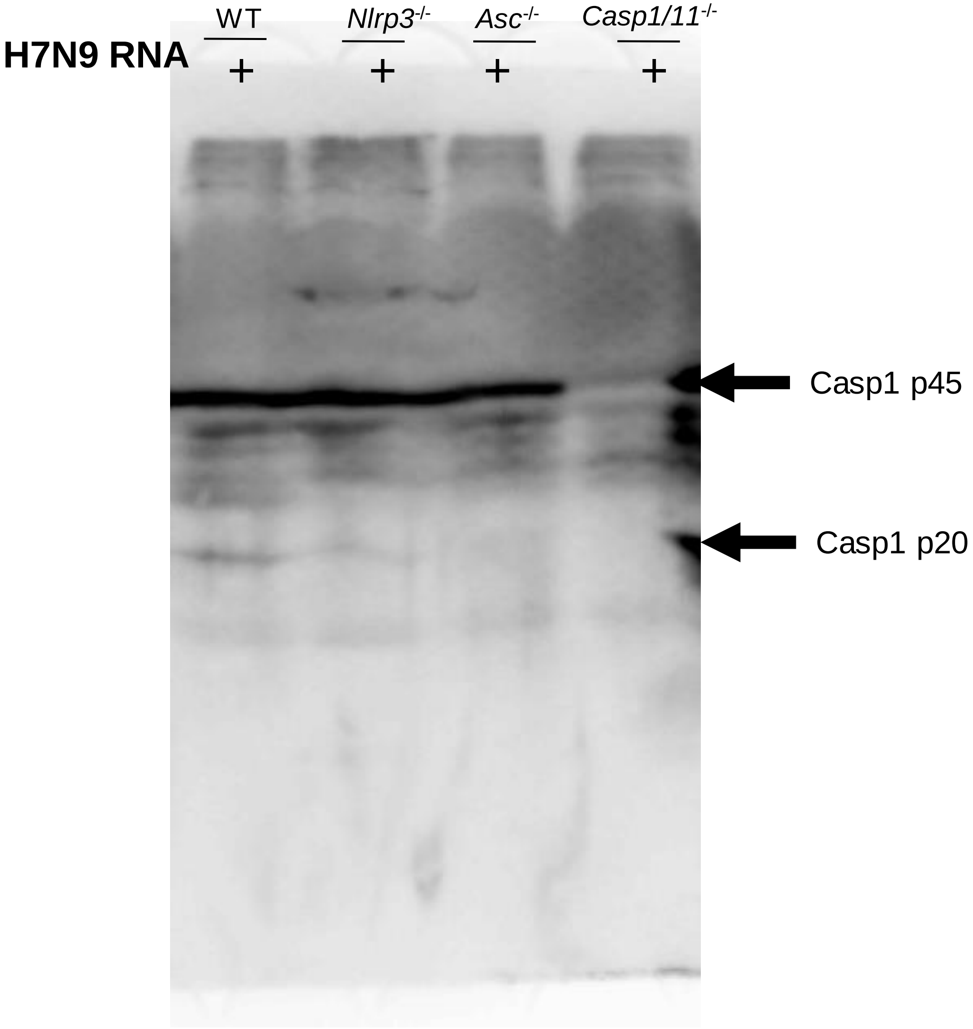

b

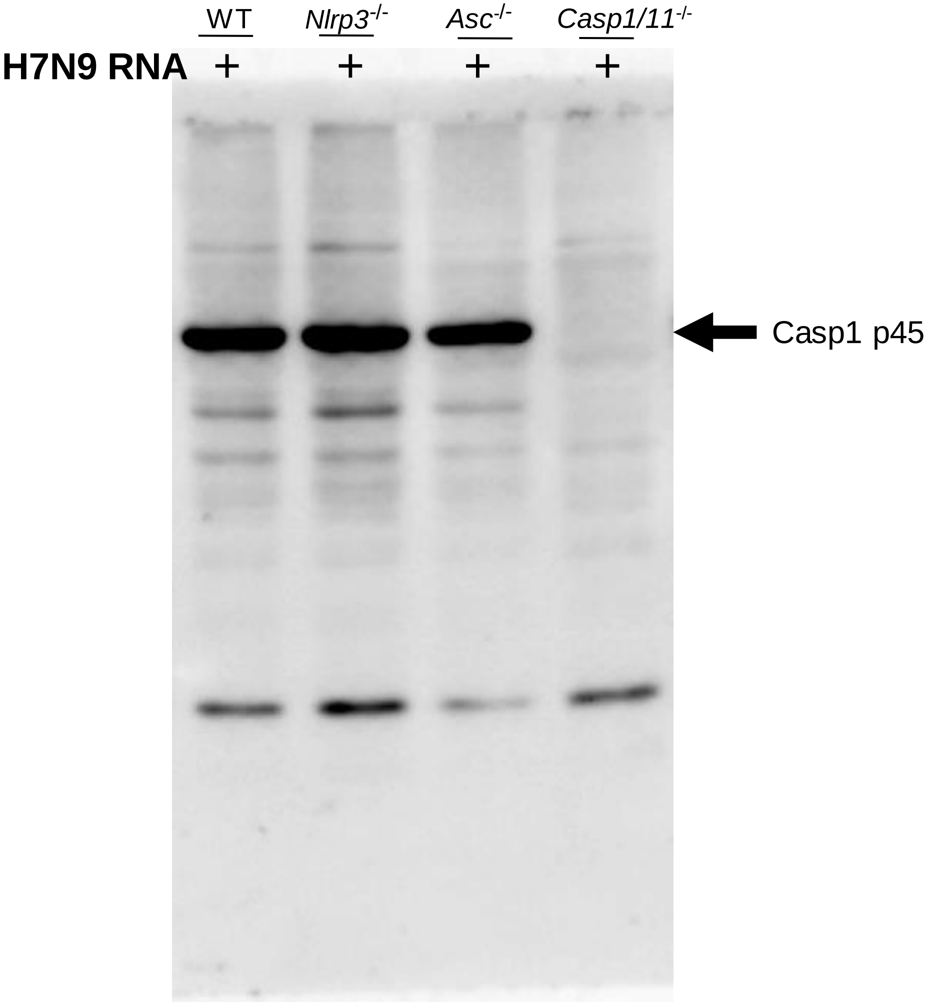

c

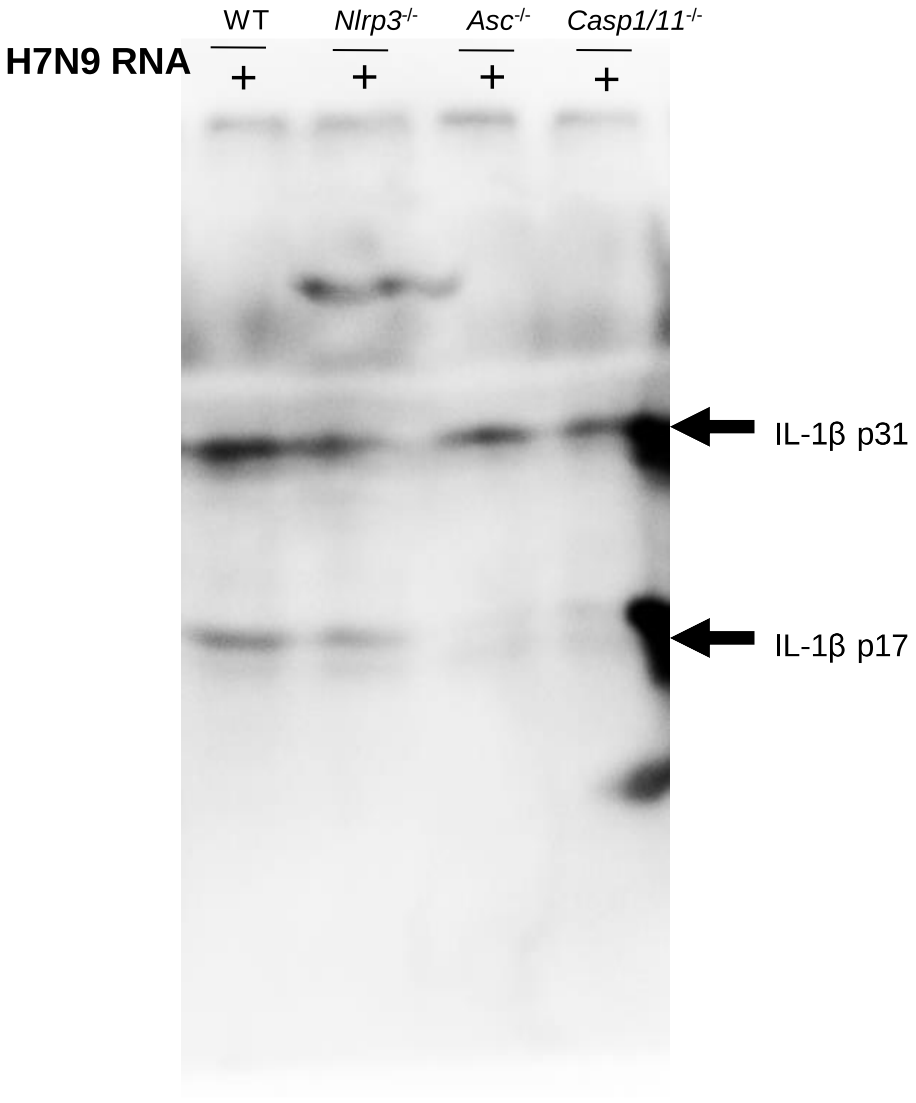
WT
Nlrp3-/-
Asc-/-
Casp1/11-/-
H7N9 RNA
+
+
+
+
IL-1β p31
IL-1β p17

d

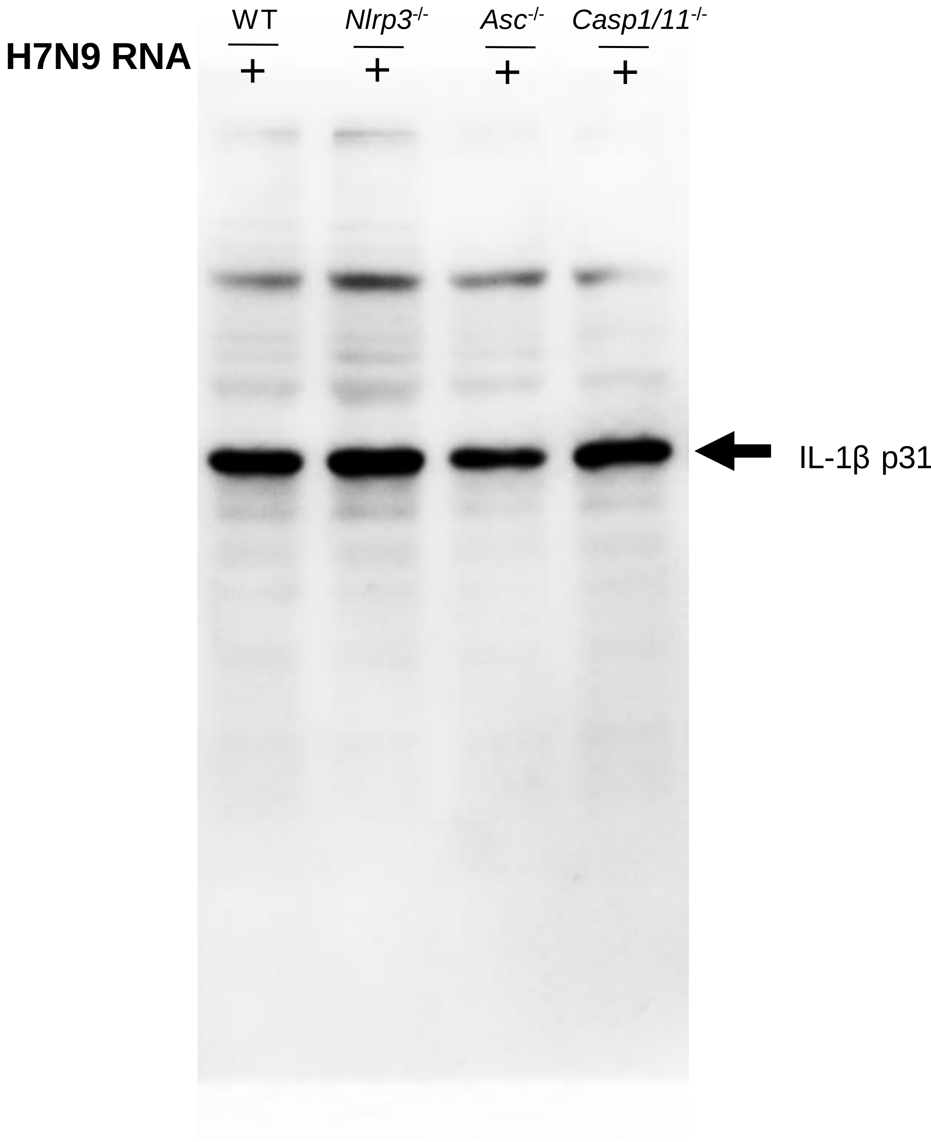
WT
Nlrp3-/-
Asc-/-
Casp1/11-/-
H7N9 RNA
+
+
+
+
IL-1β p31

e

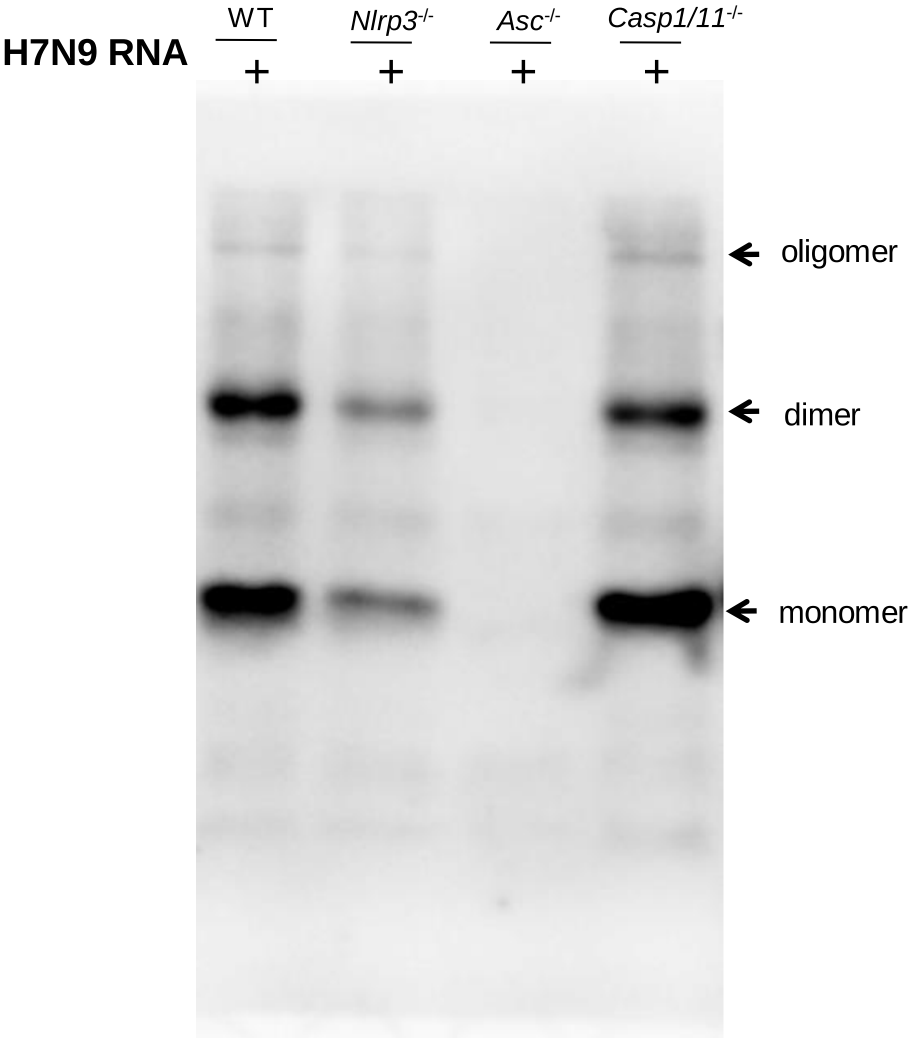
WT
Nlrp3-/-
Asc-/-
Casp1/11-/-
H7N9 RNA
+
+
+
+
oligomer
dimer
monomer

f

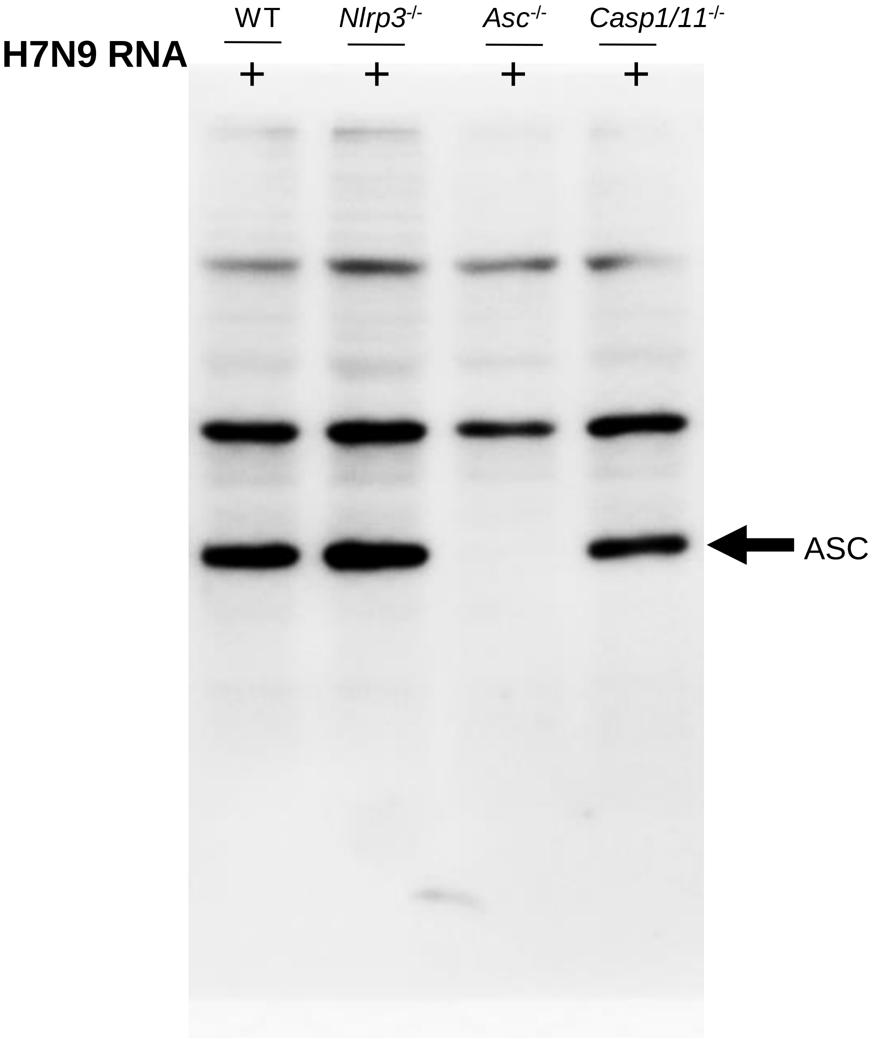
WT
Nlrp3-/-
Asc-/-
Casp1/11-/-
H7N9 RNA
+
+
+
+
ASC

g

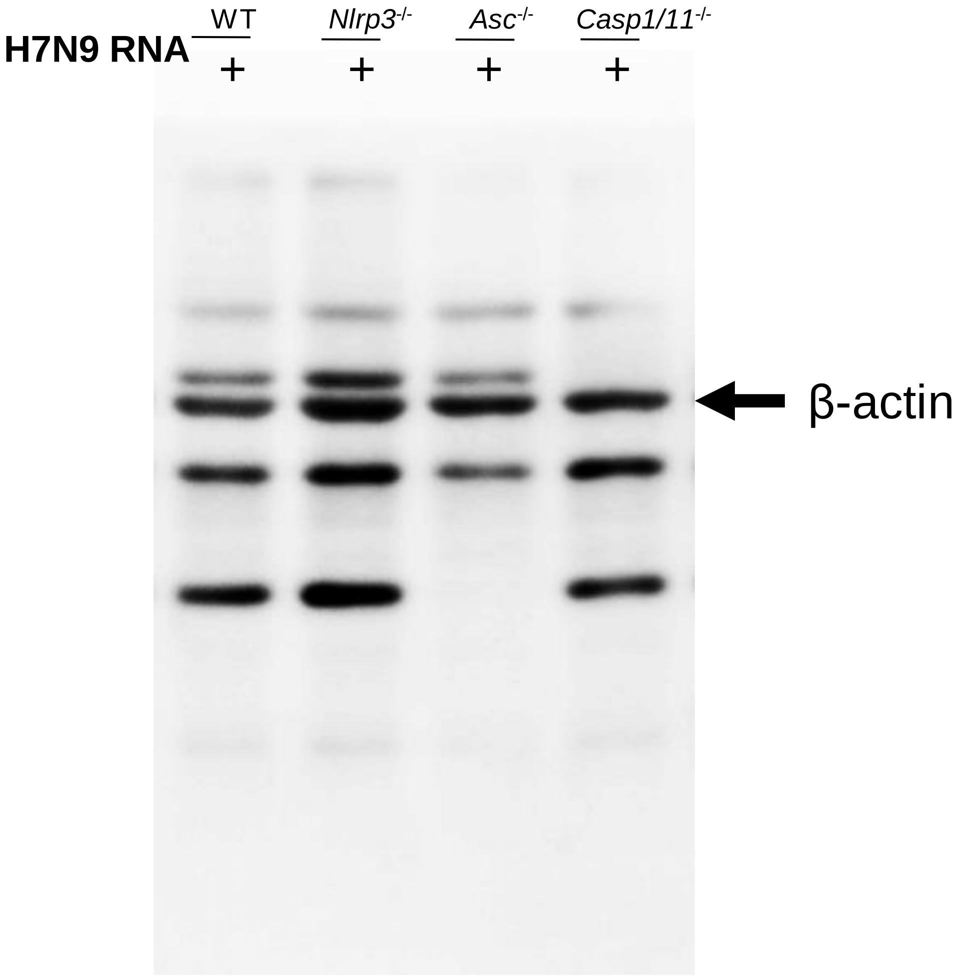

Fig. S3. NLRP3 inflammasome activation in BMDCs induced by H7N9 RNA.

The full-length gels of cleaved caspase1 and IL-1β in supernatants (S3a, c), pro-caspase1 and pro-IL-1β in cell lysates (S3b, d), ASC oligomer in DSS cross-linked pellets (S3e), ASC in cell lysates (S3f) and β-actin in cell lysates (S3g).
